# Chloroplast DNA variation in a hyperdiverse tropical tree community

**DOI:** 10.1002/ece3.5096

**Published:** 2019-03-26

**Authors:** Henri Caron, Jean‐François Molino, Daniel Sabatier, Patrick Léger, Philippe Chaumeil, Caroline Scotti‐Saintagne, Jean‐Marc Frigério, Ivan Scotti, Alain Franc, Rémy J. Petit

**Affiliations:** ^1^ BIOGECO INRA, Univ. Bordeaux Cestas France; ^2^ INRA UMR 0745 EcoFoG (Ecologie des forêts de Guyane) Kourou France; ^3^ AMAP, IRD, Cirad, CNRS, INRA Université de Montpellier Montpellier France; ^4^ INRA, UR629 Ecologie des Forêts Méditerranéennes URFM Avignon France

**Keywords:** chloroplast DNA, DNA barcoding, genetic diversity, hybridization, incomplete lineage sorting, introgression, species diversity, tropical trees

## Abstract

- We investigate chloroplast DNA variation in a hyperdiverse community of tropical rainforest trees in French Guiana, focusing on patterns of intraspecific and interspecific variation. We test whether a species genetic diversity is higher when it has congeners in the community with which it can exchange genes and if shared haplotypes are more frequent in genetically diverse species, as expected in the presence of introgression.
- We sampled a total of 1,681 individual trees from 472 species corresponding to 198 genera and sequenced them at a noncoding chloroplast DNA fragment.
- Polymorphism was more frequent in species that have congeneric species in the study site than in those without congeners (30% vs. 12%). Moreover, more chloroplast haplotypes were shared with congeners in polymorphic species than in monomorphic ones (44% vs. 28%).
- Despite large heterogeneities caused by genus‐specific behaviors in patterns of hybridization, these results suggest that the higher polymorphism in the presence of congeners is caused by local introgression rather than by incomplete lineage sorting. Our findings suggest that introgression has the potential to drive intraspecific genetic diversity in species‐rich tropical forests.

## 1 INTRODUCTION

Community structure is an important topic in population biology, yet we have only incomplete understanding of how genetic diversity is organized in a complex multispecies community. To study the composition of a community, researchers have long emphasized the number of species and to a lesser extent their relative numbers (Whittaker & Klomp, 1975). Subsequently, it was shown that the study of community structure could also benefit from a phylogenetic analysis of the component species (Cavender‐Bares, Kozak, Fine, & Kembel, 2009; Vamosi & Wilson, 2008). Yet, all these approaches still typically ignore intraspecific variation. This is unfortunate because it is increasingly clear that a multilevel approach is needed to study biological diversity (Larsson, 2001; Wilcox, 1984). Moreover, it is now well established that the genetic diversity of dominant species can feed back on the whole community (Bolnick et al., 2011; Crutsinger et al., 2006). Hence, a full analysis of community structure should integrate functional and phylogenetic intraspecific and interspecific components (Bolnick et al., 2011; Pavoine & Izsák, 2014). Whereas a few population genetic studies have sampled and analyzed multiple species simultaneously (Petit et al., 2003; Taberlet et al., 2012), they have not focused on the genetic diversity of entire communities. Performing such a study would provide a unique opportunity to investigate shared genetic variation across species and clarify its origin. It could also help identify new avenues to understand the evolution of communities.

Closely related species can share genetic characteristics if insufficient time has elapsed since speciation for divergence to have taken place through genetic drift or mutation (Neigel & Avise, 1986). The sharing of genetic attributes among species can also be caused by gene flow, which can extend beyond species boundaries as a consequence of hybridization and introgression (Arnold, 1997; Mallet, 2005; Mallet, Besansky, & Hahn, 2015). However, distinguishing introgression from incomplete lineage sorting remains a critical task in evolutionary studies (Joly, McLenachan, & Lockhart, 2009). At the scale of an entire community, the goal is not to understand the origin of genetic similarities in a particular species pair but to look at general trends across species and genera, taking advantage of the sampling of multiple independent lineages within which patterns of polymorphism and of shared genetic variation can be explored. The approaches that need to be implemented in such a context necessarily differ from those used for investigating the origin of genetic exchanges in one particular species pair or species group.

A first possibility to distinguish between incomplete lineage sorting and introgression as a source of intraspecific variation in a given community is to investigate whether genetic diversity is influenced by the presence of congeners. Local presence and relative abundance of congeneric species have long been known to influence hybridization rates (e.g., Cannon & Lerdau, 2015; Focke, 1881; Hubbs, 1955; Natalis & Wesselingh, 2013). Following hybridization, introgression, defined as the movement of genes from a donor species into the gene pool of a recipient species, should typically increase the diversity of the recipient species (Arnold, Bulger, Burke, Hempel, & Williams, 1999; Currat, Ruedi, Petit, & Excoffier, 2008). Hence, a species, genetic diversity should be higher, on average, if it lives close to congeners with which it can exchange genes. In contrast, in the absence of introgression, genetic diversity is not predicted to be higher in the presence of congeners. Whereas introgression is often reported to increase intraspecific genetic diversity (Anderson, 1949,1953; Rieseberg & Wendel, 1993), especially in hybrid zones (e.g., Barton & Hewitt, 1985, Govindaraju, Dancik, & Wagner, 1989), attempts to evaluate the effect of the presence of congeners on intraspecific genetic diversity have only been investigated in a few individual target species (e.g., Valencia‐Cuevas et al., 2015; Valencia‐Cuevas, Piñero, Mussali‐Galante, Valencia‐Ávalos, & Tovar‐Sánchez, 2014). Ideally, studies of the effect of the presence of congeners on genetic diversity should account for the species' life‐history traits known to influence the rate of introgression (Du, Petit, & Liu, 2009; Duminil et al., 2007).

A second way to distinguish between incomplete lineage sorting and introgression is to investigate whether links exist between interspecific sharing of genetic variants and intraspecific genetic diversity. Introgression should result both in the percolation of alleles across species and in an increased genetic diversity in the recipient species. As a consequence, if introgression is involved, an association is expected between interspecific sharing of genetic information and intraspecific variation. In contrast, no such relationship is expected if allele sharing originates from shared ancestry. Hence, focusing simultaneously on allele sharing and on genetic diversity could provide further evidence on the mechanism involved.

**Figure 1:**
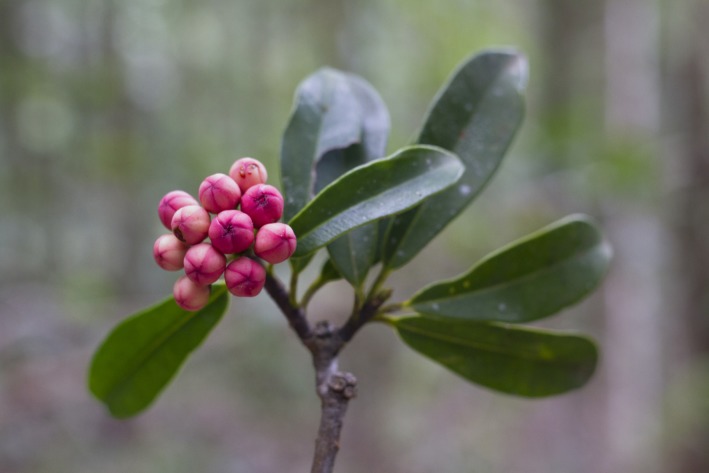
Flowering stem of *Symphonia globulifera* (Clusiaceae), one of the 704 tree species identified in the Piste St Elie long‐term research plot, located in the tropical rain forest of French Guiana. This species has at least one congener in the study plot, as 80% of the species, and its seeds are dispersed by animals, as 87% of the species. Photograph courtesy of Hadrien Lalagüe

Tropical tree communities are well suited for studying the relationship between intra‐ and interspecific variation. First, they have particularly high specific and generic diversity (Cannon & Lerdau, 2015; Condit et al., 2005; Figure 1). Second, hybridization is particularly frequent in long‐lived woody plants (Ellstrand, Whitkus, & Rieseberg, 1996; Fazekas et al., 2009; Grant, 1981; Mallet, 2005; Petit & Hampe, 2006). Yet, such studies are confronted with operational constraints: Multiple individuals of numerous species must be sampled, reliably identified and consistently scored using suitable molecular markers. Indeed, idiosyncratic hybridization barriers can make generalizations across taxa complicated (Arnold, 1997; Focke, 1881; Grant, 1981; Mallet, 2005; Whitney, Ahern, Campbell, Albert, & King, 2010), requiring the study of many species to detect general trends. Interestingly, prior investigations have demonstrated the feasibility of barcoding large numbers of tropical trees at chloroplast DNA sequences (Gonzalez et al., 2009,2010; Kress et al., 2009,2010; Pei et al., 2011). Chloroplast DNA (cpDNA) is predominantly maternally inherited in angiosperms (Harris & Ingram, 1991; Petit & Vendramin, 2007). Thus, it is especially prone to introgression, as shown by a neutral model of the process (Currat et al., 2008; Du et al., 2009; Petit & Excoffier, 2009). Hence, cpDNA could be used to estimate the extent of intraspecific genetic diversity and haplotype sharing across species, provided that errors in identifying species of voucher samples are minimized, as they might confound the analyses (e.g., Dexter, Pennington, & Cunningham, 2010).

In this study, we sampled a few individuals from a majority of the species in a well‐inventoried species‐rich tree community in French Guiana and sequenced a single noncoding cpDNA marker from each individual tree. We explore whether species with congeners in the community tend to have higher genetic diversity than species lacking congeners. We further test whether this holds regardless of species seed dispersal mode, as seed dispersal is the life‐history trait known to be most strongly associated with cpDNA variation in trees (Duminil et al., 2007). We then investigate the distribution of chloroplast haplotypes across species to test whether shared variation among species is associated with high intraspecific variation, as expected in the case of introgression.

## 2 MATERIALS AND METHODS

This study was conducted at the Piste de St Elie research station, in northern French Guiana (5°18′N, 53°30′W). Annual rainfall at the site ranges from 2,500 to 4,000 mm, with a marked dry season between August and November and a short drier period in March. Elevation ranges between 10 and 50 m above sea level. The vegetation is mainly lowland tropical rainforest. Several permanent plots of various shapes and sizes (from 0.04 to 12 ha) have been established at the site for studying tree species diversity (Madelaine et al., 2007; Sabatier et al., 1997). About 13,500 trees with diameter at breast height (dbh) ≥10 cm and 3,300 trees with 2–10 cm dbh have been permanently marked, positioned, and identified to species or morpho‐species level in an area covering ca. 21 ha. Most plots were first inventoried between 1989 and 1992, and then, twelve 1‐ha plots were surveyed again in 2003 (Madelaine et al., 2007). During these inventories, and regular visits, nearly 26% of all trees were sampled for identification at the Herbier IRD de Guyane, where voucher specimens are deposited. The remaining trees were identified in the field. To date, 704 species of trees from 238 genera have been recorded in this site.

For the present study, we sampled a total of 1,903 trees representing 433 species and 38 morpho‐species, 199 genera and 52 families. We focused on species for which several individuals had been found. Voucher specimens of 1,343 of these trees (71% of the total) are deposited at the Herbier IRD de Guyane. From each tree, we collected either fresh leaves (272 trees) or cambium tissue (1,631 trees), which were dried in silica gel following Colpaert et al. (2005). Plant species were classified as having autochorous, anemochorous, hydrochorous, zoochorous, or mixed dispersal syndromes (Howe & Smallwood, 1982; Pijl van der, 1972). There were only few species classified in each of the first three categories, so they were combined to compare genetic diversity of species with abiotic and biotic modes of seed dispersal. Species that had mixed abiotic and biotic seed dispersal strategies were classified in the biotic category, to contrast species having a purely abiotic seed dispersal strategy with species having at least partly a biotic seed dispersal strategy.

### 2.1 Molecular analysis

Total genomic DNA was extracted from dried material using an Invisorb DNA Plant HTS 96 kit as recommended by the manufacturer (Invitek GmbH, D13125, Berlin, Germany). When mixing the plant powder with the extraction buffer resulted in a gel, we added Digest‐Eur 1X (Eurobio, 91,953, Les Ullis, France) to 50% of final volume, incubated the mixture at room temperature for 20 min, and then raised the temperature to 65°C. The target cpDNA sequence was amplified by PCR following Shaw et al. (2005), using the primers *trn*H^GUG^ (Tate & Simpson, 2003) and *psb*A (Sang, Crawford, & Stuessy, 1997). In some cases, when PCR amplification failed, PCR was repeated up to three times with a more diluted (1:50–1:800) DNA matrix. An ABI 3,730 capillary sequencer (Applied Biosystems Inc., Warrington, UK) was used for the sequencing. Each PCR product was sequenced in both forward and reverse directions.

### 2.2 Sequence editing and alignment

In order to check the quality, clean and edit sequences, we used an in‐house bioperl script (Stajich et al., 2002). The complementary strands were assembled and sequences aligned by eye, using CODONCODE ALIGNER v 2.0.6 (CodonCode Corp.) and BIOEDIT v7.0.5.3 (Hall, 1999).

### 2.3 Data analysis

We identified all haplotypes taking into account insertion/deletions, inversions, and substitutions. To limit the effects of sequencing errors on estimated polymorphism rates, we considered that two sequences represent different haplotypes if they differ by at least two nucleotides, except when all individuals of a given species share a single nucleotide that distinguishes them from all other species. Species for which only one individual was successfully sequenced (26) were discarded from calculations of the percentage of polymorphic species. The resulting dataset contained sequences for 446 species from 195 genera. For the analysis of haplotype sharing, we considered all species belonging to genera for which at least two species had been analyzed (355 species distributed in 80 genera). To estimate the overall proportion of shared haplotypes, we considered each of the haplotype present in each of these 355 species, and checked whether it was present (shared) or not (private) in at least another congeneric species.

We used Fisher's exact test to compare proportions between different categories in two‐way tables. In each genus for which at least eight species had been studied, we used binomial probabilities to estimate the probability of having as many or as little observed polymorphic species, assuming a uniform probability of cpDNA polymorphism across species. Similarly, in each of these genera, we used binomial probabilities to estimate the probability of having at least as many or as little observed cases of pairs of species sharing at least one haplotype, assuming a uniform probability of cpDNA sharing across all congeneric species pairs.

### 2.4 Identification errors and their consequences

Identification errors are a clear concern in this type of study, especially if they are biased toward one category (e.g., species‐rich genera). Therefore, we adopted a parsimonious approach, carefully rechecking the sequences and botanical identifications of individuals that had haplotypes shared with another congeneric species according to preliminary analyses.

## 3 RESULTS

### 3.1 cpDNA variation

We obtained usable sequences of the noncoding cpDNA marker *trn*H‐*psb *from 1,681 trees sampled in the Piste de St Elie research station. These trees belong to 198 genera and 472 species (average of 3.6 individuals per species, range: 1–11). From these 1,681 sequences, we could distinguish 502 haplotypes. No haplotype was found to be shared between genera but 65 haplotypes were shared between two or more species from the same genus.

### 3.2 cpDNA polymorphism in the presence of congeners

Among the 446 species for which we had sequenced at least two individuals, 89 (20%) belong to genera for which a single species had been found in the study site (called group 1 species) and 357 (80%) belong to 106 genera for which at least two species (2–34) had been found in the study site (group 2 species). Fifty‐six (13%) of the analyzed species have an abiotic dispersal syndrome and 390 (87%) a biotic dispersal syndrome, with species of group 1 being more frequently abiotically dispersed than those of group 2 (25% vs. 10%, *p* = 0.0003).

The overall rate of cpDNA polymorphism is 26% (115/446). As predicted, among the 56 abiotically dispersed species, rates of polymorphism are higher in species from group 2 than from group 1 (26% vs. 0%, *p* = 0.008, Table 1). Among the 390 biotically dispersed species, the trend is weaker but in the same direction (29% vs. 16%, *p* = 0.03, Table 1). Sample sizes cannot account for these differences as the mean number of individuals sequenced per species is very close in group 1 and group 2 species (3.8 and 3.6, respectively). Hence, observed rates of cpDNA polymorphism are greater in the presence of congeners regardless of the dispersal mode, but especially so for abiotically dispersed species.

**Table 1:** Proportions of polymorphic species in group 1 (no congeners) and group 2 (congeners present) as a function of the mode of seed dispersal

Dispersal mode	Group 1	Group 2	*p*‐Value
Abiotic	0% (0/22)	26% (9/34)	0.008
Biotic	16% (11/67)	30% (97/323)	0.03
All	12% (11/89)	30% (106/357)	0.001

Note

*p*‐Values (based on Fisher's exact test) refer to the probability of the difference between values for species from group 1 and group 2 arising from chance only.

We then used a binomial model to investigate how cpDNA variation differs from the general trend in each of the 10 most locally speciose genera. In seven of the 10 genera, the proportion of polymorphic species is higher than average (115/446), and significantly so in three genera (*Eschweilera*, *Inga* and *Protium*), whereas it is never significantly below average (Table 2).

**Table 2:** Rates of species polymorphism within genus and binomial probability of having a number of polymorphic species equals to or lower (*P* ^−^) or equals to or higher (*P^+^*) than that observed, assuming a uniform rate of polymorphism across species (significant values underlined)

Genus	# Species	# Polymorphic	*P* ^−^	*P^+^*
*Talisia*	8	3	0.91	0.34
*Tovomita*	8	1	0.88	0.91
*Eschweilera*	10	7	0.35	0.004
*Ocotea*	11	3	1.00	0.57
*Eugenia*	11	2	0.43	0.82
*Protium*	11	6	0.69	0.04
*Sloanea*	10	2	0.99	0.77
*Licania*	16	7	0.50	0.09
*Pouteria*	19	4	0.97	0.76
*Inga*	21	13	0.43	0.001

### 3.3 cpDNA polymorphism and haplotype sharing

The overall proportion of shared haplotypes is 35% (155/438). This proportion is significantly higher in polymorphic species than in monomorphic species (44% [89/201] vs. 28% [66/237], *p* = 0.0004), showing that patterns of cpDNA variation and of haplotype sharing across species are not independent. For the ten largest genera, patterns of haplotype sharing across species are highly heterogeneous: In seven genera, pairs of species share haplotypes less frequently than average (173/1,262), and significantly so in five genera (*Tovomita*, *Eugenia*, *Licania*, *Pouteria,* and *Protium*), whereas only in the genus *Inga* do pairs of species share haplotypes significantly more frequently than average (Table 3).

**Table 3:** Shared variation within genus and binomial probability of having a number of species pairs sharing haplotypes equals to or lower (*P^‐^*) or equals to or higher (*P^+^*) than that observed, assuming a uniform rate of haplotype sharing across all species pairs (significant values underlined)

Genus	# Species pairs	# Species pairs sharing haplotype(s)	*P* ^−^	*P^+^*
*Talisia*	28	2	0.24	0.91
*Tovomita*	28	0	0.02	1.00
*Eschweilera*	45	8	0.84	0.27
*Ocotea*	66	8	0.44	0.70
*Eugenia*	66	0	0.00006	1.00
*Protium*	78	5	0.035	0.99
*Sloanea*	91	13	0.64	0.48
*Licania*	136	9	0.007	1.00
*Pouteria*	190	11	0.0004	1.00
*Inga*	253	71	1.0000	<10^−8^

## 4 DISCUSSION

We selected the Piste de St Elie research station in French Guiana for this work. In this site, botanical inventories started in 1986 and taxonomical efforts have continued ever since, so it is now one of the relatively few long‐term sites where tropical forests “have been studied intensively enough to generate comprehensive species lists” (Dick & Kress, 2009). More than 16,800 trees belonging to 704 taxa have been identified in this place to date. The mean number of species per genus recorded in the study site (2.9) is consistent with results obtained in other tropical rainforests (Condit et al., 2005; Dick & Kress, 2009), corroborating previous estimates showing that in tropical forest, local coexistence of trees with allospecific congeners is the norm rather than the exception (Cannon & Lerdau, 2015). Hence, the Piste St Elie site appears to be highly suitable for studying patterns of genetic variation within and among closely related species at the scale of a community.

Our strategy was to examine numerous species in order to characterize broad patterns of variation, at the expense of more thorough sampling within species (about 3–4 individuals per species, on average). Sampling more individuals per species would have likely reduced the number of analyzed species, as many species are very rare. We reasoned that even small samples can provide useful information on levels of population genetic variation, as suggested earlier (Nei, 1978).

Considering these low sample sizes, the levels of cpDNA sequence polymorphism were remarkably high in the study site (26% of polymorphic species). We found that species are more likely to be polymorphic when other congeneric species had been identified in the study site. The presence of congeneric species that coexist locally with a given species is a prerequisite for hybridization and introgression, which ultimately result in the enrichment of the target species gene pool. This reasoning had been used earlier to explain increased genetic diversity in sympatric parts of congeneric species ranges (e.g., Valencia‐Cuevas et al., 2014). Generalizing across taxa in a community, we suggest that the observed difference in the rate of cpDNA polymorphism in species with or without congeners can be explained in the same way. Moreover, we found that the trend of higher genetic diversity in the presence of congeners is boosted in species with abiotic mode of dispersal. Such species typically experience reduced gene flow by seeds (Duminil et al., 2007), resulting in increased conspecific spatial aggregation (Seidler & Plotkin, 2006). Maternally inherited cpDNA markers should be more readily introgressed in these species, in line with models predicting an inverse relationship between intraspecific gene flow and introgression (Currat et al., 2008; Du et al., 2009). Hence, the particularly high contrast of cpDNA polymorphism observed in the presence or absence of congeners in abiotically dispersed species further supports our inference that introgression is at least in part responsible for the higher polymorphism in the presence of congeners.

We found 65 unique cpDNA sequences (i.e., haplotypes) shared among at least two species and 437 haplotypes restricted to a single species. Interestingly, the distribution of these haplotypes is not independent of the level of intraspecific variation. Haplotypes detected in polymorphic species are more frequently shared with congeners than haplotypes from monomorphic species. This observation fits well with the hypothesis of introgression, which should result not only in an increased diversity in the recipient species but also in an increased rate of haplotype sharing between the recipient and donor species. If sharing of haplotypes among species was caused instead by the retention of ancestral variants, we would instead expect that haplotypes from monomorphic species (i.e., fixed for a single haplotype) would be preferentially shared, assuming that frequent alleles are on average older than less frequent ones (Slatkin & Rannala, 2000).

In such a study, a number of pitfalls could complicate inferences. We cannot exclude that some unknown biogeographic process results in a pattern whereby species belonging to rapidly diversifying lineages and hence more likely to coexist with congeners have higher levels of intrapopulation variation. However, this hypothesis cannot account for the observed relationship between cpDNA diversity and haplotype sharing. In principle, identification errors could produce the same pattern than introgression (haplotype sharing and increased “intraspecific” diversity). In general, in tropical forests, botanical work is complicated by the difficulty of collecting fertile material from trees during inventories (Dexter et al., 2010), an important problem because sterile material is more difficult to identify to the species level. However, long‐term studies such as this one maximize the chances of observing fertile material and hence of correctly identifying species. Moreover, focusing on a single forest, as was done here, should reduce the risk of incorrectly splitting species, compared to studies involving sampling over broad areas. During the study, we adopted a conservative approach, questioning the results of each sample whose haplotype did not fit with that of the other individuals of its species by rechecking sequences and vouchers or collecting new ones. Importantly, we found no evidence for reduced taxonomic resolution in large genera, as haplotype sharing per species pair was lower than average in most of these genera. Hence, identification mistakes are unlikely to account for the high proportion of polymorphic species in genera represented by more than one species in the community. This finding also illustrates an important point: the lack of support for a general snowball effect of introgression in the largest genera. Such an effect is expected if pairs of species that cannot mate directly are able to exchange haplotypes via “bridging” species, resulting in extensive mating networks, as reported in tropical mangrove trees (Cerón‐Souza et al., 2010). What we find instead is that several species‐rich tree genera appear to exchange haplotypes among species at a reduced frequency, perhaps because of the increased selective pressure against maladaptive hybridization when many congeners are present (i.e., reinforcement).

Relying on multiple nuclear markers could help confirm species status using multilocus assignment methods. Such approaches have been used in previous population genetic investigations in the region and elsewhere, demonstrating unequivocally the role of introgression in specific genera, typically by relying on geographic patterns to infer introgression (e.g., Bänfer et al., 2006; Du et al., 2009; Duminil, Caron, Scotti, Cazal, & Petit, 2006; Jackson, Potts, & Vaillancourt., 1999; Kamiya, Harada, Tachida, & Ashton, 2005; Palme, Su, Palsson, & Lascoux, 2004, Scotti‐Saintagne et al., 2012).

No study so far had examined effects of introgression on an entire species‐rich forest community. The present study aimed at filling this gap. Altogether, our findings suggest a link between intra‐ and interspecific diversity in tropical forests, with the presence of congeners generating increased intraspecific diversity through increased opportunities for hybridization and introgression. This particular effect of species diversity on genetic diversity had so far not been considered in efforts to conceptualize and explain biodiversity (cf. Vellend, 2005; Vellend & Geber, 2005). Given that introgression can facilitate the transfer of adaptive traits (Chapman & Abbott, 2010; Kim et al., 2008; Kim & Rieseberg, 1999; Martinsen, Whitham, Turek, & Keim, 2001; Whitney, Randell, & Rieseberg, 2010), an increase in baseline levels of genetic diversity through introgression should boost the potential for adaptation and diversification (Arnold, 1997; Field, Ayre, Whelan, & Young, 2008; Grant, 1981; Lewontin & Birch, 1966; Seehausen, 2004).

More studies on a nearly entire local flora, focusing on different markers and traits, are needed, despite the inherent difficulties of assembling such datasets, especially in terms of field work and taxonomic expertise. Such investigations could help make key advances in the long‐standing debate on the importance of introgression in evolution (Anderson, 1949,1953; Barton, 2001; Dowling & Secor, 1997; Rieseberg, 1995; Rieseberg & Wendel, 1993; Stebbins, 1959). They also illustrate the benefits of an extended analysis of community structure by considering biodiversity also at the intraspecific level, thereby further integrating ecology and evolution.

## CONFLICT OF INTEREST

None declared.

## AUTHOR CONTRIBUTIONS

HC, AF, JFM, and CSS designed the study. HC, JFM, CSS, and DS performed the field sampling. JFM and DS identified all trees in situ or using vouchers. HC and PL performed the sequencing. HC, PC, and JMF checked and edited the DNA sequences. HC, IS, AF, and RJP analyzed output data. RJP wrote the first draft of the manuscript, and all authors contributed to revisions.

## Data Availability

Sequences have been deposited in the R‐SYST databank (https://github.com/r-syst/databases/tree/master/r-syst::plants) and NCBI under accession number KX247940–KX249593. The list of studied species, including genus size, seed dispersal syndrome and cpDNA variation, is available at https://github.com/r-syst/databases/tree/master/r-syst::plants. In this list, chloroplast haplotype designation is consistent within each genus.
